# Drumming-associated anthrax incidents: exposures to low levels of indoor environmental contamination

**DOI:** 10.1017/S0950268818001085

**Published:** 2018-07-04

**Authors:** E. Bennett, I. M. Hall, T. Pottage, N.J. Silman, A.M. Bennett

**Affiliations:** Public Health England, Porton Down, Salisbury, Wiltshire, SP4 0JG, UK

**Keywords:** anthrax, drumming-related, *Bacillus anthracis*, indoor contamination

## Abstract

Two fatal drumming-related inhalational anthrax incidents occurred in 2006 and 2008 in the UK. One individual was a drum maker and drummer from the Scottish Borders, most likely infected whilst playing a goat-skin drum contaminated with *Bacillus anthracis* spores; the second, a drummer and drum maker from East London, likely became infected whilst working with contaminated animal hides.

We have collated epidemiological and environmental data from these incidents and reviewed them alongside three similar contemporaneous incidents in the USA. Sampling operations recovered the causative agent from drums and drum skins and from residences and communal buildings at low levels. From these data, we have considered the nature of the exposures and the number of other individuals likely to have been exposed, either to the primary infection events or to subsequent prolonged environmental contamination (or both).

Despite many individual exposures to widespread low-level spore contamination in private residences and in work spaces for extended periods of time (at least 1 year in one instance), only one other individual acquired an infection (cutaneous). Whilst recognising the difficulty in making definitive inferences from these incidents to specific residual contamination levels, and by extending the risk to public health, we believe it may be useful to reflect on these findings when considering future incident management risk assessments and decisions in similar incidents that result in low-level indoor contamination.

## Introduction

In the first decade of the 2000s, a series of human anthrax incidents were reported in the UK and the USA [1–5]. Of these five incidents, four involved single anthrax cases, three inhalational and one gastrointestinal; in the fifth incident, there were two cutaneous anthrax cases in the same family. All incidents were related directly, or indirectly, to the exposure to contaminated animal skins used for drum making and drumming. Aside from occupational infections, there has not been such a series of human anthrax incidents reported since in the West, despite the continuing popularity of drumming and drum making with animal skins. It is likely that hide skins and drums contaminated with low levels of anthrax will continue to be imported into the UK and other countries even though simple measures can be taken to reduce this risk by the treatment of hides [6]. Indeed, contaminated animal skins from anthrax-endemic countries are also thought to have been the source of *Bacillus anthracis* spores in contaminated heroin, responsible for the 2010/2011 and 2012/13 European anthrax outbreaks in injecting drug users [7, 8]. Although very rare in the West, there have also recently been a couple of inhalational anthrax incidents where it has been unclear as to how the cases acquired their infections; one involving a vaccinated soldier and one involving a man who had been on holiday in four US states where anthrax is enzootic [9, 10].

In all incidents reviewed here, low levels of indoor environmental contamination with *B. anthracis* were reported, and in some of them, large groups of people were potentially exposed. For the 2006 and 2008 UK inhalational anthrax incidents, we have presented the background epidemiology and environmental sampling data. For the US incidents, we have reported the sampling data for context where available. We have also considered the number of other individuals likely to have been exposed to either (or both) the primary infection incidents or to subsequent environmental contamination.

## Materials and methods

The microbiological and epidemiological data from environmental investigations of two recent UK fatal inhalational incidents in the Scottish Borders and East London have been reviewed using published reports and unpublished data [1, 4]. Both incidents involved single drumming- or drum making-associated anthrax cases, where subsequent environmental contamination was reported as low level. For comparison, a literature search was undertaken in Embase and Scopus using search terms anthrax OR anthracis AND drum* for other drumming-related anthrax infections and identified the three other recent drumming-associated anthrax investigations from the USA, where low-level environmental contamination was reported. Low-level contamination is defined here as recorded levels just above detectable limits. These were an inhalational anthrax incident in New York City [3], a gastrointestinal anthrax incident in New Hampshire [5, 11] and a cutaneous anthrax incident in Connecticut [2]. For each incident, the numbers of people potentially exposed to reported spore levels have been estimated by referring to the authors’ descriptions of those individuals identified as potentially exposed and offered prophylaxis in the reference publications and, in the instance of the contaminated village hall in the Scottish Borders, by making estimates of the likely number of users over the period in which the hall was in use before its closure.

For the microbiological results reported for the 2006 and 2008 UK incidents, the detection limit by culture was deemed to be one spore; using sponge or air samplers, the sample is diluted in 5 ml of diluent and 50 µl plated. This means that each colony on an agar plate represents a total of 100 organisms in the original sample. Because of this dilution effect, samples with between 1 and 100 spores will be PCR-positive but culture-negative.

For determining the numbers of spores present on the rug, Public Health England data indicate that with a clean preparation of spores, the nucleic acid extraction efficiency is ~10% and recovery of spores from samples with a high dust or dirt content is, at best, 50%. If these caveats are applied to the samples from the rug, then the spore numbers present may be as high as 2 × 10^4^ in total. The distribution is unknown.

## Results

### Incident 1: fatal inhalational anthrax, Scottish Borders, 2006

In July 2006, a man in the Scottish Borders died from atypical anthrax [4]. The case had regularly attended local drumming workshops, predominantly at a village hall since early 2006. The recovery of *B. anthracis* from blood culture was confirmed 1 month after death. The case's home tested negative for *B. anthracis* after an extensive environmental sampling campaign, but three premises were subsequently determined by culture and PCR to be contaminated: (i) a residential property in England, home to a drum maker/teacher and his partner from August 2006; (ii) the village hall, where the case had taken part in a drumming class the day before becoming ill; and (iii) a farmhouse in the same village, where the class teachers had stayed before moving in August 2006. A summary of the sampling results is shown in Table 1.

**Table 1.** PCR and culture results from samples taken from buildings in Scotland and England 2006

Location	PCR-positive No. (% of total)	Culture-positive No. (% of total)	Total samples tested
Case's home	0	0	103
Village hall	10 (52.6)	2 (10.5)	19
Village farmhouse	5 (12.2)	0	41
Village farmhouse (garage floor)	1 (7.1)	1 (7.1)	14
House, England (floors)	12 (16)	2 (2.6)	75
Van	2 (12.5)	0	16
Drums	3 (12)	2 (8.0)	25
Skins	2 (33.3)	1 (16.6)	6
Total	35 (11.7)	8 (2.7)	299

In the home of the drum maker/teachers, widespread low-level contamination was detected by PCR in most rooms and in a van [12]. *Bacillus anthracis* was isolated by culture in samples from the floor, a rug under stored drums, from drums and a drum skin. Calculation of the numbers of spores present within a vacuum sample taken from the rug indicated that up to 2 × 10^4^ spores/m^2^ could have been present. The isolate obtained was deemed by expert opinion to be the same strain as the isolates from the case [12]. The culture-positive drum skin had been brought into the UK in the previous year from Guinea, where anthrax is endemic [4]. Background air samples collected during the entire sampling operation were negative for viable spores and spore DNA, suggesting that any re-aerosolisation, and thus aerosol exposure to airborne *B. anthracis* during normal activities, would be insignificant [12].

In the village hall, PCR-positive samples were found throughout: in the kitchen, toilets, main hall, other ancillary rooms and vacuum cleaner. *Bacillus anthracis* was isolated by culture from chairs in a second room, from the floor and from brooms used in the main room. These strains were indistinguishable by variable number tandem repeat (VNTR) genotype analysis from the one isolated from the case [13]. Again, all air samples were negative for viable spores and spore DNA.

In the village farmhouse where drums were stored, PCR-positive samples were found throughout the ground floor and in vacuum cleaners. In the garage, viable spores were detected on the floor and DNA found on drum storage and work surfaces.

As the tolerable level of *B. anthracis* was at the time defined as no detectable spores, and a detectable spore was defined as one detected by culture, all premises were decontaminated in which viable spores were detected [4].

#### Estimate of individuals exposed

An estimate of the number of individuals potentially exposed to *B. anthracis* spore contamination in this incident, and the other following incidents, is shown in Table 2.

**Table 2.** Summary estimates of the number of individuals exposed to primary exposure and subsequent low-level residual *Bacillus anthracis* environmental contamination

Incident	Nature of primary exposure^a^	Numbers potentially exposed to primary exposure^b^	Numbers potentially exposed^c^ to secondary environmental (residual) contamination	Levels of secondary environmental spore contamination	Duration of secondary exposure	Number of cases resulting from any exposure	Estimated total of individuals potentially exposed to primary exposure and secondary contamination
Inhalational anthrax (fatal), Scottish Borders, 2006	Playing *B. anthracis*-contaminated drum at a drumming workshop	~74 (attendees at same drumming classes as fatal case)	~400^d^ (subsequent hall users)	333–1000 (CFU per 30 ml sample) (widespread contamination)	5 months (village hall)	0	478
			~4 (in private residences)	33–66 (CFU per 30 ml sample) (widespread contamination)	Up to 1 year in one residence		
Inhalational anthrax (fatal), London, 2008	Drum making and manipulating contaminated animal hides	1 (drum making colleague)	NA	No environmental contamination (contamination level on drums and hides ~1000 CFU)	Not known	0	1
Gastrointestinal anthrax, New Hampshire, USA, 2009	Playing a contaminated drum or exposure to contaminated food	79 (attendees at drumming workshop)	5 (living or working at event site)	20–44 (total CFU)^e^ (two drums: 300 and 171 total CFU)	3½ weeks (community centre)	0	84
Inhalational anthrax, New York City and Pennsylvania, 2006	Manipulating/shaving contaminated animal hides for drum making	4	4 (same individuals as previous column)	No quantitative sampling reported but widespread contamination	Not known	0	8
Cutaneous anthrax, Connecticut, 2007	Manipulating/shaving contaminated animal hides for drum making	0	4 (household members; including one anthrax case)	<10^f^ (total CFU) (household, widespread contamination) (Shed workshop samples indicated ‘heavy growth’ by visual inspection)	Not known	1	4
Estimated total		**158**	**417**			**1**	**575**

a Assumed to be most likely source of exposure following investigation.

b Assuming there was a ‘one-time’ exposure.

c Estimates based on reported information.

d Number based upon estimate of hall users over a 5-month period.

e The limit of detection by culture was 20 CFU per sample.

f Culture limits of investigations not given.

**Drumming classes and workshops:** Classes were regularly held at the village hall and less frequently at other local venues. As well as participants’ own drums, more than 20 imported drums, including the known contaminated one, were available for use [4]. Seventy-four people were identified as having had contact with the drums, either as drumming class members or workshop attendees (Table 2). The last workshop in a neighbouring town took place 3 days before the case became ill and involved 20–30 people.

**General use of the village hall:** The hall was in use for 5 months between the time the case died and hall closure. Contamination may have been present for a longer time; however, the hall was regularly used for drumming classes. It is estimated that around 400 individuals used the hall over this period.

**Contaminated residences:** The two drumming teachers and the parents of one lived in the village farmhouse where PCR-positive samples were recovered. It is not known for how long this residence might have been contaminated or who else might have been exposed. The second residence, where drums were stored and processed since August 2006, was also found to contain viable spores and was occupied for at least a year, until decontamination [4].

### Incident 2: fatal inhalational anthrax case, London, 2008

In October 2008, a man from Dalston, London, died from anthrax [1]. He made and played drums, and had contact with other skins and drums during drumming circle meetings [14]. Viable spores were recovered from a drum in his flat and from two animal hides in an adjacent communal locked storage area. *Bacillus anthracis* isolated from the drum was determined by genotypic studies not to be the same as that isolated from the case. Isolates from the two hides were found to be closely related to the isolate from the case and were likely identical [14]. All other samples, including air samples, from the flat were negative. Approximately 1 × 10^3^ viable spores were recovered from each of the drum and skin samples taken by vacuum filter sampling and sponge wipe [14].

**Estimate of individuals exposed:** The patient's immediate family, living in a separate flat, the main skins supplier, a person who helped with drum making and a concerned hospital staff member were all considered at some risk of exposure and were offered antibiotic prophylaxis for 60 days [1].

### Incident 3: gastrointestinal anthrax, New Hampshire, USA, 2009

In December 2009, a woman attended a drumming circle in a community centre, 1 day before onset of symptoms of gastrointestinal anthrax [5]. The case, who survived, used an animal-hide drum for the 2 h drumming circle and was confirmed with anthrax 19 days later. Three positive samples, two from drum heads and one from electrical outlets, were recovered from initial environmental testing of the centre. All air samples were negative. Wider, targeted, semi-quantitative environmental testing of the site and additional drums showed low-level contamination, with one positive sample from each of two drums and four from environmental locations in the building (Table 2) [11] indicating that aerosolisation of spores from drum heads had occurred. All isolates matched the patient's isolate by VNTR analysis using eight loci [11].

**Estimate of individuals exposed:** Eighty-four other individuals were potentially exposed to *B. anthracis*; 79 persons at the drumming circle and five who lived or worked at the event site. There were also an unknown number of individuals using the event site over nearly 3½ weeks before its closure.

### Incident 4: inhalational anthrax, New York City and Pennsylvania 2006

In February 2006, a New York City resident was diagnosed with anthrax [3]. The patient made traditional African drums by using hard-dried animal hides, lived in an apartment in Manhattan and had a drum-making studio workspace in a warehouse. *Bacillus anthracis* was cultured from the animal hides he worked with and these matched the isolate from the patient, who subsequently recovered. The evidence suggested that the patient's primary exposure to aerosolised *B. anthracis* spores resulted from scraping a contaminated hide in his workspace [15], for which the patient wore no protective equipment or protective clothing [3].

Widespread contamination was detected by culture and PCR from the patient's workspace, patient's residence and vehicle; with all isolates being of a single genotype, as determined by multi-locus VNTR molecular typing analysis (MLVA-8 genotype 1). Most samples from a contact's nearby workspace were also positive, indicating that spreading of spores had occurred [3].

**Estimate of individuals exposed:** Three close contacts that had regularly visited or performed similar work in his same workspace. These three, plus a fourth contact, had been in the workspace whilst hides were being shaved and swept-up during routine cleaning, the week before the patient became ill.

### Incident 5: two cutaneous anthrax cases associated with drum making, Connecticut, 2007

A drum maker presented to hospital in August 2007 with an eschar on his arm with lymphangitic spread and *B. anthracis* was detected by PCR [16]. Four days later, the drum maker's child presented with cutaneous anthrax.

Six of 25 drum heads, 15 of 35 hides, all 16 shed samples (many indicating heavy growth – unquantified in report) [2], the car boot and 18 of 72 house samples were culture-positive for *B. anthracis* (multiple-locus VNTR genotype 1) and were indistinguishable from clinical samples of the drum maker and child [16]. Three of the 18 household samples grew *B. anthracis*, indicating secondary contamination with viable spores. Subsequent sampling of the home indicated widespread contamination.

**Estimate of individuals exposed:** Despite widespread, low-level contamination of the house, anthrax did not develop in any of the three other household members.

## Discussion

In this paper, we have presented new microbiological and epidemiological data from two UK drumming-related fatal inhalational anthrax incidents and also considered the published reports of three other contemporaneous-related incidents in the USA.

Across these multiple exposure events, we have estimated that around 400 individuals had been exposed to environmental *B. anthracis* surface contamination, ranging from 33 to 1000 CFU per sample. From these, only one anthrax case was reported (apart from the point cases) – a cutaneous case in a close family member sharing a contaminated residence [2]. This is despite exposure to widespread, low-level contamination present for extended periods of time (up to a year in one instance) in private residences or in work spaces. This finding would seem to agree with conclusions drawn from the testing of environmental sites (both indoor and outdoor) associated with *B. anthracis* during the 1980s and 1990s, which considered the risk of contracting anthrax from contaminated soils and other environmental materials to be ‘exceedingly low’, with the chances of inhalational or gastrointestinal ‘next to nil’ [17]. We also estimate that at least 150 other people, who shared the same environments as the anthrax cases at the primary exposure events, were potentially exposed to aerosolised spores, yet there were no other cases arising from these exposures (Table 2).

Public perception of *B. anthracis* is that it is highly infective, but in reality humans are considered to be ‘moderately resistant’ to infection [18]. Studies have shown that immune responses have been detected in the absence of disease in both immunologically naïve individuals exposed to low levels of contamination in the Amerithrax incidents [19] and in individuals routinely exposed occupationally [20, 21]. Historically, the UK industrial anthrax incidence rates during the 19th and early 20th centuries were low for cutaneous infections and even lower for inhalational infections [22–24]. More recent occupational studies report similar evidence [20, 21, 25, 26].

There is also reasonable epidemiological evidence that even substantial exposure to aerosolised *B. anthracis* spores is necessary before the risk of inhalational anthrax becomes significant [18, 22–25]. During the ‘Amerithrax’ attacks, a large number of individuals were potentially exposed to widespread, large amounts of aerosolised *B. anthracis* spores, but only a small number of infections (inhalational or indeed cutaneous) were recorded. In the Brentwood Mail Processing and Distribution Centre, only four inhalational cases were reported, despite surface vacuum samples identifying environmental spore concentrations up to 9.7 × 10^6^ CFU/g. These concentrations suggest a large initial aerosol exposure to the workers [27]. In the AMI building in Florida, only two inhalation cases were reported despite widespread distribution of spores over many floors [28]. In the New York City area, only a few cases of cutaneous anthrax, and no inhalation cases, were reported [29].

Those inhalational anthrax cases reviewed for this paper were likely exposed to a different (higher) level of risk than the individuals who were subsequently exposed to the resulting contaminated environments. Playing drums or shaving and manipulating contaminated skins would likely have produced sufficient energy to aerosolise bound spores and make them available to be inhaled [30] and cases would likely have had prolonged exposure to infectious material. Perhaps cases were exposed to particularly high levels of spores or to a ‘mega particle containing a very large number of spores’ [31] – possibly a contaminated skin flake from the drum surface [30]. Nevertheless, other people would have played contaminated drums during classes and been exposed to aerosolised spores, but did not succumb to infection.

Some of the infected individuals had health conditions that might have made them more susceptible to infection than the ‘average’ person: the Scottish Borders case was in remission from acute myeloid leukaemia – complicated historically with pneumonia [32]; the East London case had been successfully treated for tuberculosis, and the gastrointestinal case in New Hampshire had parasitical infections, including hookworm [5]. There are other instances of increased susceptibility to inhalational anthrax infection reported in people with underlying medical conditions or impaired immune systems [9, 33–36]. A proportion of the exposed population would therefore, perhaps, be more susceptible to disease than others, with likelihood of infection dependent on age and health status, as well as route and duration of exposure. The infectious, and moreover lethal, dose for a (highly) susceptible individual would likely be lower than the median infectious or lethal dose for an immunocompetent ‘healthy’ individual; however, we do not know what this dose might be.

A number of assumptions have been made when reviewing these outbreaks and incidents. It is likely that the number of potentially exposed individuals has been underestimated, since we cannot be certain of the number of people who may have had contact with contaminated items or environments. In the Scottish Borders, for example, the contaminated drum skin had been in the UK for over a year and played, as a drum, at other times; in the New Hampshire incident, the drum had been used by others regularly for 10–15 years. More importantly, perhaps, though is that the actual levels of environmental contamination involved in each incident will never be known. Routine cleaning in some environments, the village hall, for example, may have reduced the levels of contamination before sampling took place and, as it is, sampling efficiency (recovery of spore material) is generally considered to be between 10% and 20% [37]. It is also difficult to draw generalisable quantitative conclusions across the five incidents; sampling and analysis techniques differed between organisations and in none of the sampling campaigns was rigorous statistical sampling employed – the remit of the sampling was primarily to determine a source and identify organism presence using targeted sampling. In future investigations, more robust, quantitative assessments would serve to refine these estimates and allow perhaps for more optional approaches to decontamination to be considered [38].

To the same extent, with the limitations outlined above in mind, we cannot make robust inferences about the risk to public health from exposure to the recorded spore levels. It may never become clear what factors caused the cases to become infected and ill, when others, sharing the same environments, did not. It is likely that it was a combination of an unusually high spore concentration, possibly a dislodged skin flake from the drum skin, and inhalation/ingestion by a highly susceptible exposed person. The extent to which an individual's underlying disease or compromised immunity might influence the likelihood and course of anthrax infection, however, is largely unknown. We also do not know the reason why five infection events were reported within the space of 4 years yet none have been reported since, despite the likelihood that contaminated skins continue to be imported and handled. It is possible that this is due to human physiological tolerance to the infection through repeated exposure; Wattiau *et al.* found circulating antibodies or T lymphocytes reacting with anthrax-protective antigen in unvaccinated workers in an anthrax-contaminated wool and goat hair factory, with no recorded clinical cases. The authors also found that the extent of the individual workers’ response to anthrax spores from the environment varied enormously [21]. It could also be due to sheer bad luck on the part of those five cases or perhaps it was the success of public health messages given since these incidents having played a role in preventing further cases.

## Conclusion

These findings are from five incident investigations whose remits were to identify source, rather than rigorously statistically sampled contaminated environments. Therefore, it is difficult to infer actual levels of environmental contamination to which individuals were exposed and, by extension, what the risk was to public health. But we can suggest that the levels that were identified here were insufficient to cause clinical infection in the vast majority of individuals exposed, despite the timeframes over which they were exposed, supporting the World Health Organisation 2008 statement that humans are moderately resistant to anthrax infection.

Data from incidents such as these, despite their limitations, are findings and observations from real-life events involving individuals with their own inherent variability and risk factors. It is a great challenge to unpick the multitudinous factors of variability and uncertainty involved with anthrax infection, although science continues to progress. So, we argue, these findings can currently provide important context for wider discussions on the likelihood of human anthrax infection from indoor exposure to *B. anthracis*, and possible incident recovery options, particularly where contamination levels are low. However, in occupational settings or instances of bioterrorist activity, public health assessment and response might be different to that for naturally acquired incidents. Because of the scale or nature of the population potentially exposed, or the element of malicious intent, tolerance of residual contamination, and recommendations for post-exposure prophylaxis, might necessarily be different. Moreover, an intentional attack against a naïve population, with a modified organism or a very pathogenic strain, might have different low-dose effects, necessitating a situation-specific response. Nevertheless, in some circumstances, where an environment is contaminated with low numbers of spores, there might still be an opportunity to consider a flexible clean-up strategy; one that is proportionate to the likelihood of infection from exposure to settled spores rather than a single-response approach based on a remediation until there are no detectable spores.
